# Supplementary material for: Deep Proteomic Analysis on Biobanked Paraffine-Archived Melanoma with Prognostic/Predictive Biomarker Read-Out
Source: Cancers (Basel). 2021 Dec 3;13(23):6105. doi: 10.3390/cancers13236105 (PMC8657028; doi:10.3390/cancers13236105)
Supplement: Supplementary file 1 [file cancers-13-06105-s001.zip › Suppl.Figure_Cancers_FFPE-2021-11-24_SZL_final.pdf]

Table of contents

FIGURE S1 2
FIGURE S2 3
FIGURE S3 4
TABLE S1 5
TABLE S2 7
FIGURE S4 9

Figure S1

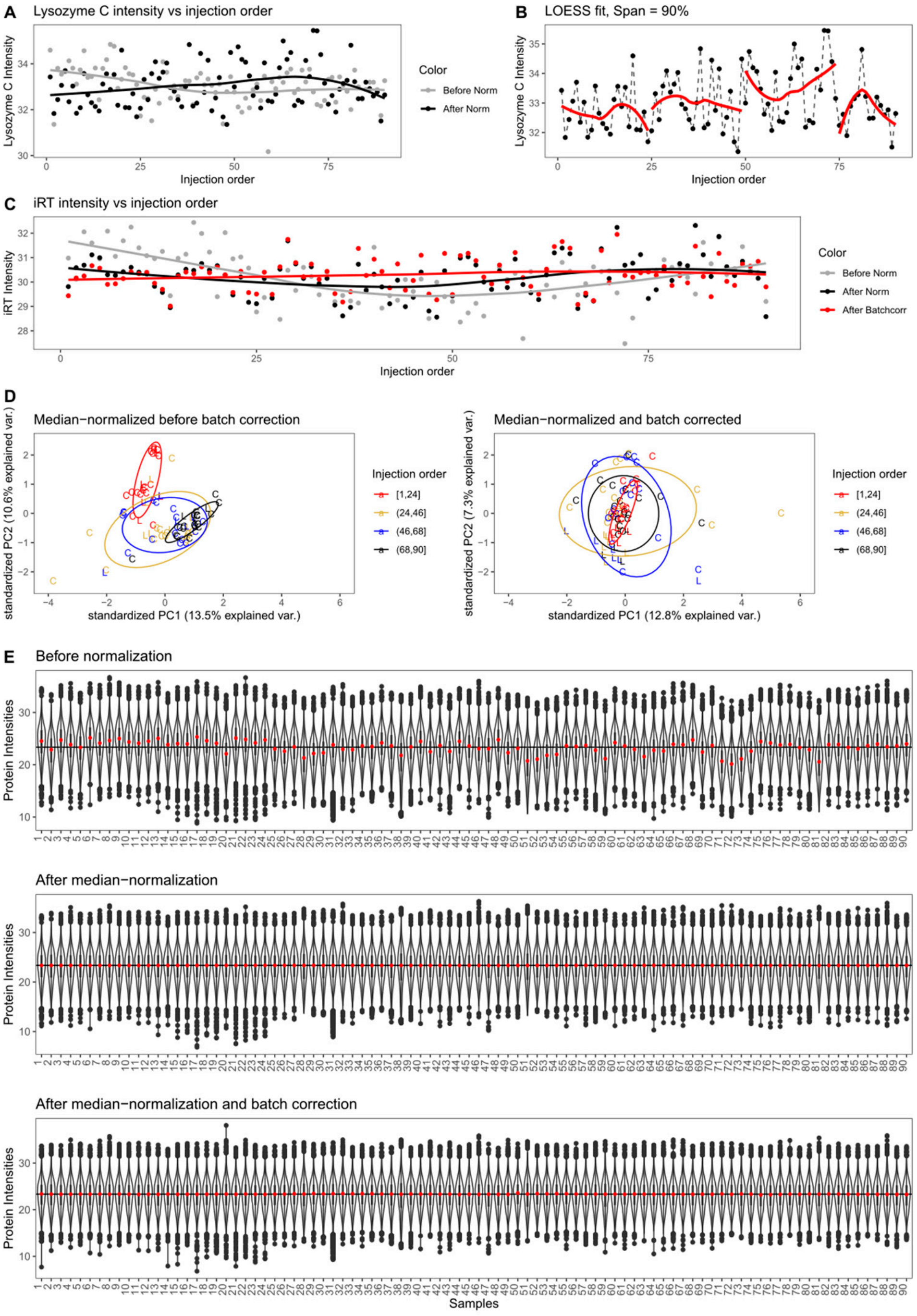

Figure S1. Quality control plots for proteomic data normalization and batch effect correction. (A) Lysozyme C (spike-in) protein intensity versus the injection order in the LC-MS/MS analysis. A continuous drift in the intensity values was observed before and after median-normalization, shown in dark grey and black respectively. (B) Continuous drift correction of Lysozyme C protein intensities based on a batch correction method implemented in proBatch. A non-linear trend (shown in red) was fitted on the intensities in each batch, which was then subtracted from all protein intensity values. (C) The iRT intensity before and after normalization, and after batch correction (shown with dark grey, black and red respectively). After batch correction, the intensities were fairly constant. (D) Principal Component Analysis (PCA) plot of the samples after median normalization. Separation based on the injection order was observed. (E) PCA plot of the samples after batch effect correction. Separation based on the injection order was no longer visible, as well as samples of lymph node origin were mainly clustering separately from samples of cutaneous origin (denoted as L and C on the plot).

Figure S2

A

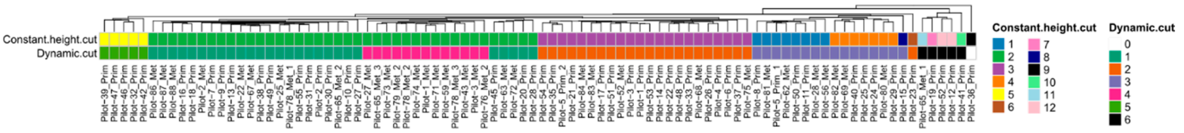

B

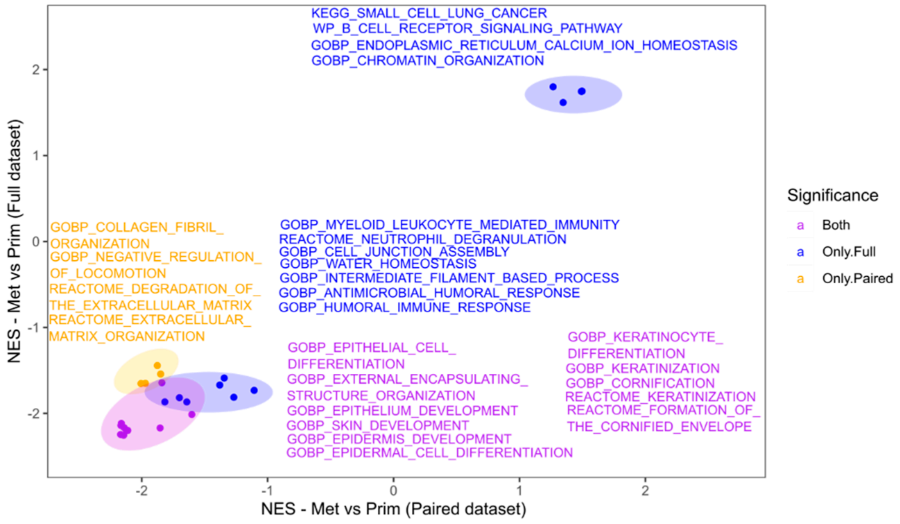

C

	All identified proteins	Proteins used in ANCOVA	Significantly upregulated proteins (p-value < 0.05 and regression coefficient > 0)	Significantly downregulated proteins (p-value < 0.05 and regression coefficient < 0)
# Proteins	**7881**	**6907**	**147**	**152**

D

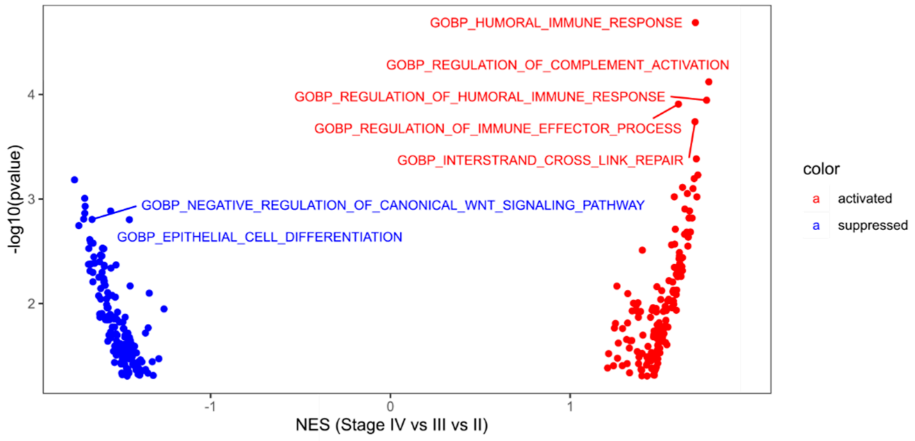

Figure S2. Addressing sample heterogeneity in our study. (A) Difference between dynamic tree cutting and tree cutting at a constant height. The samples are clustered based on their Z-score normalized protein expression table using Euclidean distance and complete linkage. Heatmap annotations show the results of tree cutting at a constant height (setting the number of clusters to an arbitrary value of 12), and the results of dynamic tree cutting. The latter method chose the optimal number of clusters automatically, was able to detect nested clusters and did not result in multiple clusters consisting of only a single sample. (B) A contrast between activated and suppressed processes in metastasis samples when comparing the 8 paired patient samples (paired t-test) or when comparing all samples (t-test between 53 primary and 37 metastasis samples). Significance was set to pGSEA FDR < 0.05 and dots are colored according to significance in the datasets. A positive NES indicates an activated gene set in metastasis samples, whereas negative NES indicates suppression of the gene set in metastasis samples. A high alignment between the results was detected, with multiple commonly significant gene sets, and when significance was not shared, the processes were still indicating the same direction (i.e., same NES sign) in both datasets. (C) Summary of ANCOVA results comparing protein expression between clinical stages II-IV. (D) Pre-ranked gene set enrichment analysis results for proteins showing linear up- and downregulation from stage II to IV. Gene sets discussed in the text are highlighted.

Figure S3

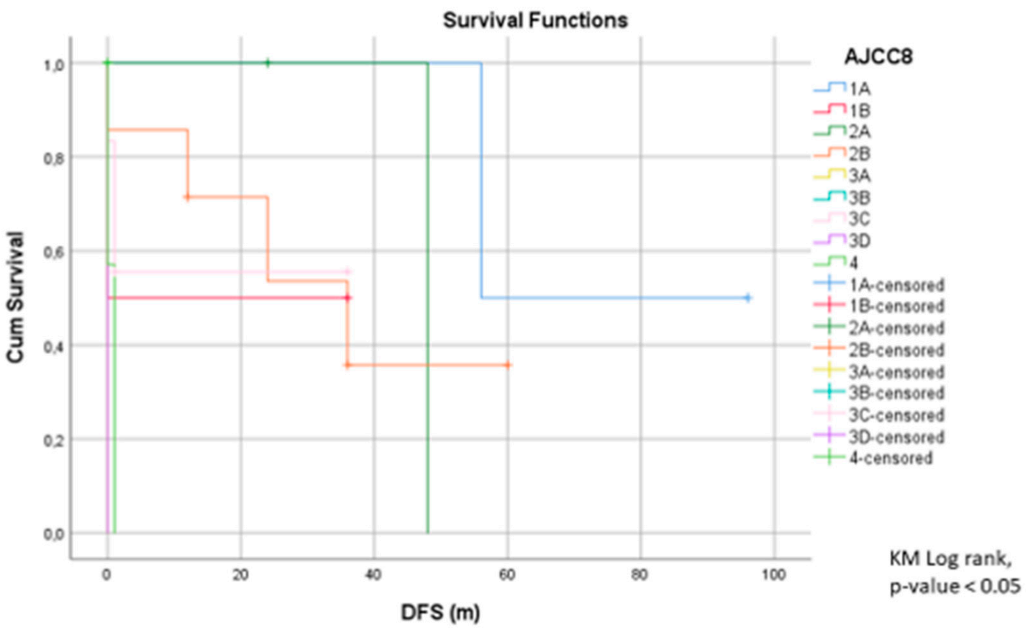

Percentiles

AJCC8	25,0% Estimate	25,0% Std. Error	50,0% Estimate	50,0% Std. Error	75,0% Estimate	75,0% Std. Error
1A			56,000	.	56,000	.
1B			,000	.	,000	.
2A	48,000	.	48,000	.	48,000	.
2B	.	.	36,000	13,301	12,000	12,749
3A	,000	.	,000	.	,000	.
3C					1,000	,894
3D	,000	.	,000	.	,000	.
4	1,000	.	1,000	.	,000	.
Overall	56,000	.	36,000	21,982	,000	.

Overall Comparisons

	Chi-Square	df	Sig.
Log Rank (Mantel-Cox)	5,277	1	,022
Breslow (Generalized Wilcoxon)	4,001	1	,045
Tarone-Ware	4,784	1	,029

Figure S3. Kaplan-Meier Survival analysis of the treated patients based on their AJCC8 classification stage during disease-free survival. /DFS is calculated in months (m), DFS – disease free survival, AJCC8 - American Joint Committee on Cancer 8th Edition/ KM Log rank, Breslow (Generalized Wilcoxon),Tarone Ware p-value < 0.05. Kaplan-Meier survival analyses and figures showing p-values, quartile values, and 95% confidence intervals were produced by IBM SPSS statistics package (26.0 version) software.

Table S1

Table S1. Fisher's exact test results for clinical and histopathological categories (borderline) significantly enriched in the detected sample clusters. /Abbreviations: Mel - melanoma, OS - overall survival, DFS - disease free survival, PFS - progression-free survival. /

Cluster	*Trait*	*P value*	*Patient Ratios*	*Odds Ratio*
1	Localisation of primary mel. – head and neck	0.0355	In Cluster: 9/26, Out of Cluster: 9/62	0.3255
1	OS – 101 to 205 (m)	0.0466	In Cluster: 7/26, Out of Cluster: 6/61	0.3010
2	Type of Samples - Primary	0.0240	In Cluster: 18/23, Out of Cluster: 35/67	0.3077
2	DFS – 11 to 30 (m)	0.0835	In Cluster: 6/23, Out of Cluster: 7/64	0.3529
2	Regression of the primary mel. - yes	0.0918	In Cluster: 8/23, Out of Cluster: 11/61	0.4174
3	Localisation of primary mel. – upper extremities	0.0069	In Cluster: 6/16, Out of Cluster: 6/72	0.1564
3	PFS – 61 to 100 (m)	0.0913	In Cluster: 4/15, Out of Cluster: 7/72	0.3016
4	Type of Samples - Metastasis	<0.0001	In Cluster: 13/13, Out of Cluster: 24/77	0.0000
4	Organ of the Samples – lymph node	<0.0001	In Cluster: 13/13, Out of Cluster: 11/77	0.0000
4	AJCC8 Stage - 3D	0.0286	In Cluster: 4/12, Out of Cluster: 6/75	0.1794
4	Breslow – 4.1 to 8 mm	0.0616	In Cluster: 6/11, Out of Cluster: 19/73	0.2982
4	Sex - male	0.0703	In Cluster: 10/13, Out of Cluster: 39/77	0.3117
4	DFS – 0 to 10 (m)	0.0778	In Cluster: 10/12, Out of Cluster: 43/75	0.2723
4	Localisation of primary mel. – lower extremities	0.0786	In Cluster: 6/12, Out of Cluster: 19/76	0.3382
5	Subtype - ALM	0.0001	In Cluster: 3/5, Out of Cluster: 0/85	0.0000

5	Localisation of primary mel. – lower extremities	0.0014	In Cluster: 5/5, Out of Cluster: 20/83	0.0000
5	Sex - female	0.0171	In Cluster: 5/5, Out of Cluster: 36/85	0.0000
5	BRAFstate - NO (cKIT mutation)	0.0568	In Cluster: 1/5, Out of Cluster: 0/83	0.0000
5	AJCC8 stage - 2A	0.0647	In Cluster: 2/5, Out of Cluster: 6/82	0.1243
5	Type of Samples – Primary mel.	0.0653	In Cluster: 5/5, Out of Cluster: 48/85	0.0000
5	PFS – 10 to 30 (m)	0.0662	In Cluster: 4/5, Out of Cluster: 29/82	0.1399
5	OS – 10 to 30 (m)	0.0662	In Cluster: 4/5, Out of Cluster: 29/82	0.1399
5	AJCC8 stage - 3C	0.0774	In Cluster: 3/5, Out of Cluster: 17/82	0.1791
6	DFS – 30 to 60 (m)	0.0342	In Cluster: 3/5, Out of Cluster: 12/82	0.1189

Table S2

Table S2. The stages of patients at diagnosis and after follow-up in Cluster 1, 2 and 4. Clinical stage based on AJCC8 classification.

Cluster 1 (n = 26)	Stage at diagnosis	Stage after follow up	Cluster 2 (n=23)	Stage at diagnosis	Stage after follow up	Cluster 4 (n=12)	Stage at diagnosis	Stage after follow up
Pilot-10	IIB	IIB	*Pilot-37*	IA	IIIA	*Pilot-27*	IIB	IV
Pilot-65	NA	NA	*Pilot-84*	IIIC	IV	*Pilot-3*	IIID	IIID
Pilot-2	IB	IV	*Pilot-68*	IV	IV	*Pilot-76*	IV	IV
Pilot-30	IIA	IV	*Pilot-33*	IV	IV	*Pilot-65*	NA	NA
Pilot-87	IV	IV	*Pilot-5*	IV	IV	*Pilot-43*	IIID	IV
Pilot-27	IIB	IV	*Pilot-35*	IIB	IIB	*Pilot-59*	IIIC	IIIC
Pilot-25	IIB	IV	*Pilot-23*	IIA	IV	*Pilot-79*	IIIC	IV
Pilot-76	IIID	IIID	*Pilot-54*	IIB	IIB	*Pilot-78*	IIID	IIID
Pilot-67	IV	IV	*Pilot-53*	IIIC	IV	*Pilot-1*	IV	IV
Pilot-31	IIB	NA	*Pilot-51*	IIB	IV	*Pilot-71*	IIIA	IV
Pilot-7	IV	IV	*Pilot-26*	IIA	IIIC	*Pilot-74*	IIIC	IV
Pilot-9	IIB	IV	*Pilot-52*	IV	IV	*Pilot-73*	IIIB	IV
Pilot-45	IIID	IIID	*Pilot-17*	IIIB	IIIB			
Pilot-86	IIIC	IV	*Pilot-4*	IIIB	IV			
Pilot-72	IIA	IV	*Pilot-14*	IV	IV			
Pilot-88	IIB	IV	*Pilot-75*	IIB	IV			

Pilot-13	IIIC	IV	*Pilot-6*	IIIC	IV			
Pilot-63	IA	IV	*Pilot-21*	IIIC	IV			
Pilot-28	IIIC	IV	*Pilot-1*	IV	IV			
Pilot-38	IIB	IV	*Pilot-22*	IIID	IIID			
Pilot-18	IIB	IV	*Pilot-48*	IIIC	IIIC			
Pilot-20	IIIB	IIIB	*Pilot-83*	IIA	IV			
Pilot-16	IIIB	IIIB	*Pilot-3*	IIID	IIID			
Pilot-55	IIIC	IIIC						
Pilot-49	IIA	IIA						
Pilot-22	IIID	IIID						

Figure S4

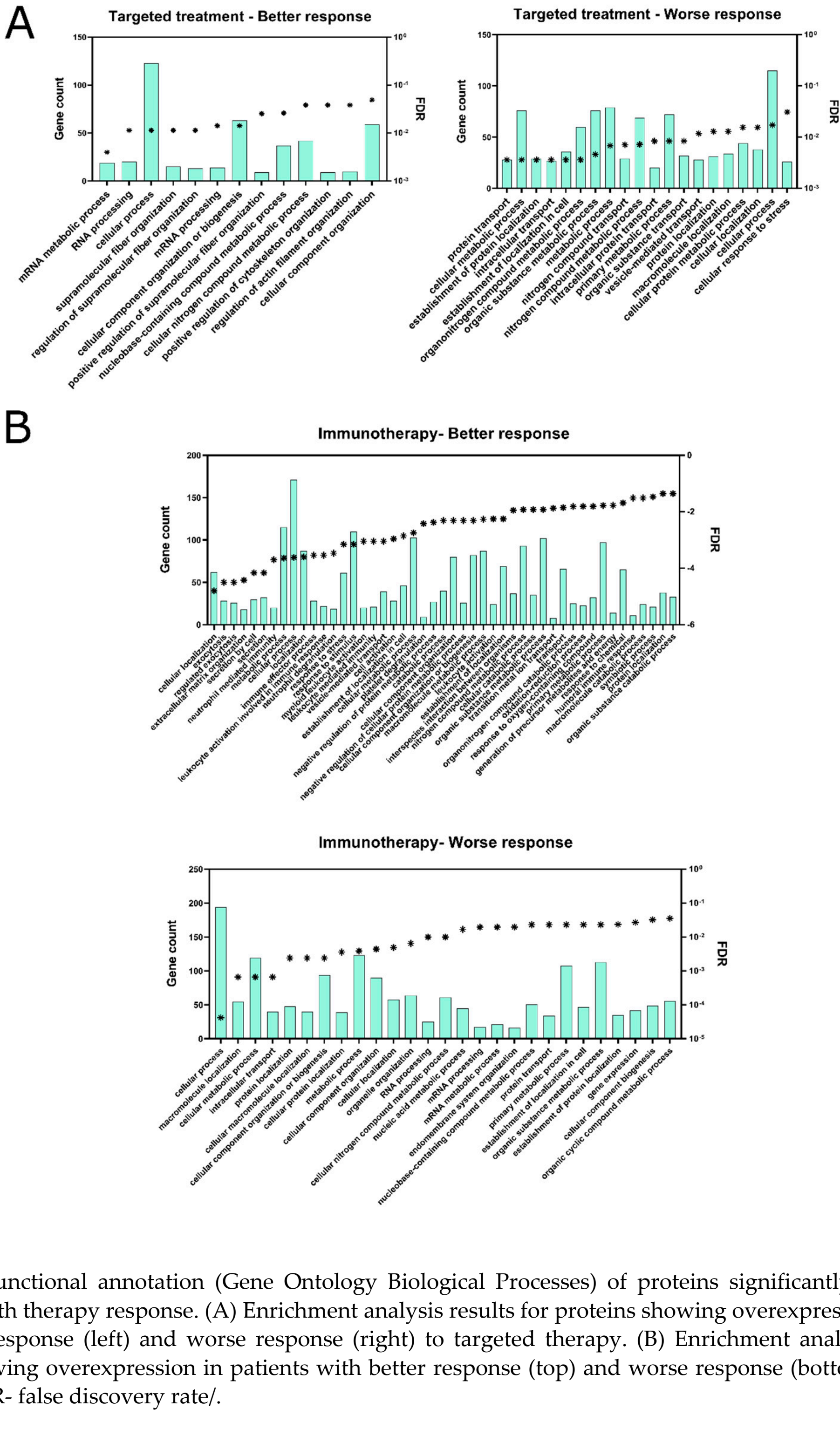

Figure S4. Functional annotation (Gene Ontology Biological Processes) of proteins significantly (FDR < 0.05) associated with therapy response. (A) Enrichment analysis results for proteins showing overexpression in patients with better response (left) and worse response (right) to targeted therapy. (B) Enrichment analysis results for proteins showing overexpression in patients with better response (top) and worse response (bottom) to targeted therapy. /FDR- false discovery rate/.
